# Internal Volumes of Pharmaceutical Compendial Induction Port, Next-Generation Impactor With and Without Its Pre-separator, and Several Configurations of the Andersen Cascade Impactor With and Without Pre-separator

**DOI:** 10.1089/jamp.2019.1590

**Published:** 2020-07-28

**Authors:** Daryl L. Roberts, Frank Chambers, Mark Copley, Jolyon P. Mitchell

**Affiliations:** ^1^ Applied Particle Principles, LLC, Hamilton, Virginia.; ^2^ Inhalytic Ltd., Kent, United Kingdom.; ^3^ Copley Scientific Ltd., Nottingham, United Kingdom.; ^4^ Jolyon Mitchell Inhaler Consulting Services, London, Ontario, Canada.

**Keywords:** cascade impactor, induction port, internal volume, pre-separator

## Abstract

***Background:*** Determination of aerosol aerodynamic particle size distributions (APSD) from dry-powder inhalers (DPIs), following quality control procedures in the pharmacopeial compendia, requires that the flow through the measurement apparatus, comprising induction port, optional pre-separator, and cascade impactor, starts from zero on actuation of the inhaler, using a solenoid valve to apply vacuum to the apparatus exit. The target flow rate, governed by the inhaler resistance, is reached some time afterward. Understanding the behavior of the DPI design-specific flow rate-rise time curve can provide information about the kinetics of the initial powder dispersion in the inhaler and subsequent transport through the APSD measurement equipment. Accurate and precise measures of the internal volume of each component of this apparatus are required to enable reliable relationships to be established between this parameter and those defining the flow rate-rise time curve.

***Methods:*** An improved method is described that involves progressive withdrawal of an accurately known volume of air from the interior passageways of the apparatus-on-test that are closed to the outside atmosphere. This approach is applicable for determining internal volumes of components having complex internal geometries. Filling some components with water, along with volumetric or gravimetric measurement, has proven valuable for the induction port and for checking other measurements.

***Results:*** Values of internal volume are provided for the USP (United States Pharmacopeia)/PhEur (European Pharmacopoeia) induction port, the Next-Generation Impactor (NGI™) with and without its pre-separator, and various Andersen 8-stage cascade impactor configurations with and without their pre-separators.

***Conclusion:*** These data are more accurate and precise, and therefore update those reported by Copley et al.

## Introduction

Pharmacopeial methods^(^^1,2)^ for measuring the size distribution of particles emitted by dry-powder inhalers (DPIs) in the product quality control environment represent a trade-off between mimicking realistic patient use and the need to keep the methodology for particle size determination as simple as possible to optimize accuracy, precision, and robustness.^(3)^ Size fractionation by multi-stage cascade impaction is the only methodology that is capable of determining the physiologically important aerodynamic particle size distribution (APSD), while also capturing enough material for quantitative determination by chemical assay of the active pharmaceutical ingredient(s) in those size fractions.^(4)^ In realistic patient use, the patient must inhale before aerosol can be emitted from a passive DPI, which currently comprises the majority of marketed products of this class of inhaler.^(3)^ Therefore, the compendial methods require the inhaler to be attached to the cascade impactor before the test begins; then, the flow is started and is increased as quickly as possible from zero to a standard flow rate; and finally, the flow is turned off after a specified volume of air passes through the DPI.^(1,2)^

Problems arise in this testing strategy because of the following limitations:

(1) the physical processes associated with the particle transport process through the cascade impactor and subsequent size fractionation based on differing particle inertia are defined only for steady air flow,^(5)^ and not during the start-up and shut-down phase of the procedure;
(2) the relationship between flow rate and release of particles from the passive DPI is different for every inhaler design and is largely associated with the flow resistance of the device^(6)^; therefore, the air flow start-up transient affects each DPI differently.

The shut-down phase is likely unimportant, because by then, after sampling the 4 L of volume specified in the pharmacopeial compendia for this performance test,^(1,2)^ all of the dispersed particulate will have been transported through, and collected by the measurement apparatus, whichever cascade impactor design is used.^(7)^ However, the situation is entirely different at start-up, where the precise trajectory of flow rate with elapsed time can potentially influence both powder dispersion into the aerosol and subsequent transport of the aerosol through the apparatus.^(3)^

Hitherto, there has been a sparsity of data to guide the user community on the start-up flow kinetics for passive DPI testing following pharmacopeial procedures. The primary purpose of a recently undertaken multi-laboratory investigation, sponsored by the European Pharmaceutical Aerosol Group (EPAG), was to establish the variation in volumetric flow rate as a function of time from initiation of sampling by various aerosol collection apparatuses currently in widespread use for DPI performance testing.^(8,9)^ These studies have confirmed the intuitively obvious dependence of the flow transient duration on the internal volume of the test system as well as on the flow resistance of the inhaler. However, the internal volumes of the various measurement apparatuses (e.g., cascade impactor, pre-separator, United States Pharmacopeia [USP]/European Pharmacopoeia [PhEur] induction port) must be well defined, to establish quantitative relationships between the parameters governing the start-up kinetics.

In a preliminary study, published in 2005 by Copley et al.,^(10)^ a technique was developed for measuring the volume of the test systems, one that relied on the fact that pressure (*P*) times volume (*V*) is a constant in a closed system (ideal gas law). The pressure was measured in a closed system that included the impactor components along with an air-tight syringe whose plunger could be withdrawn to specified volume increments. The *P*-*V* data were analyzed to deduce the unknown volume of the test system by using the several different syringe volumes that generated a range of internal pressure values. In this way, the intricacies of the internal geometry of the impactor components were included in the measurement of the total volume, without having to be completely understood. Since then, the need for a nonlinear analysis of the data became clear as opposed to the linear approach that was undertaken for the 2005 study. Separately, the reported internal volume of the USP/PhEur induction port, stated therein to be 85 mL, came under scrutiny because this value is 25% larger than the range of values between 66 and 68 mL provided by other investigators. In consequence, the 85-mL value is now regarded as erroneously large. Given this background, the EPAG researchers therefore decided that better experimental methods and data analysis methods were needed for measuring internal volumes of the several components of the most widely encountered apparatuses defined in the pharmacopeial compendia for the determination of inhaler aerosol APSD. The purpose of this study is, therefore, twofold:

(a) to provide a more robust methodology to determine apparatus internal volumes relevant to the EPAG study previously mentioned^(8,9)^ both accurately and precisely;
(b) to update and correct errors in the record provided by the 2005 study.^(10)^

## Materials and Methods

The inhaler performance test apparatuses evaluated for internal volume were the Next-Generation Impactor (NGI™) and the Andersen 8-stage nonviable cascade impactor (ACI), both as described in the PhEur^(1)^ and USP,^(2)^ including the USP/PhEur induction port and appropriate pre-separators. The ACI has several configurations for testing DPIs at the specified flow rates of 28.3, 60, and 90 L/min,^(11,12)^ and each of these were therefore included. Further, many of these configurations are pertinent to the apparatus configurations evaluated in the EPAG DPI flow rate-rise time study.^(8,9)^

**FIG. 1.**
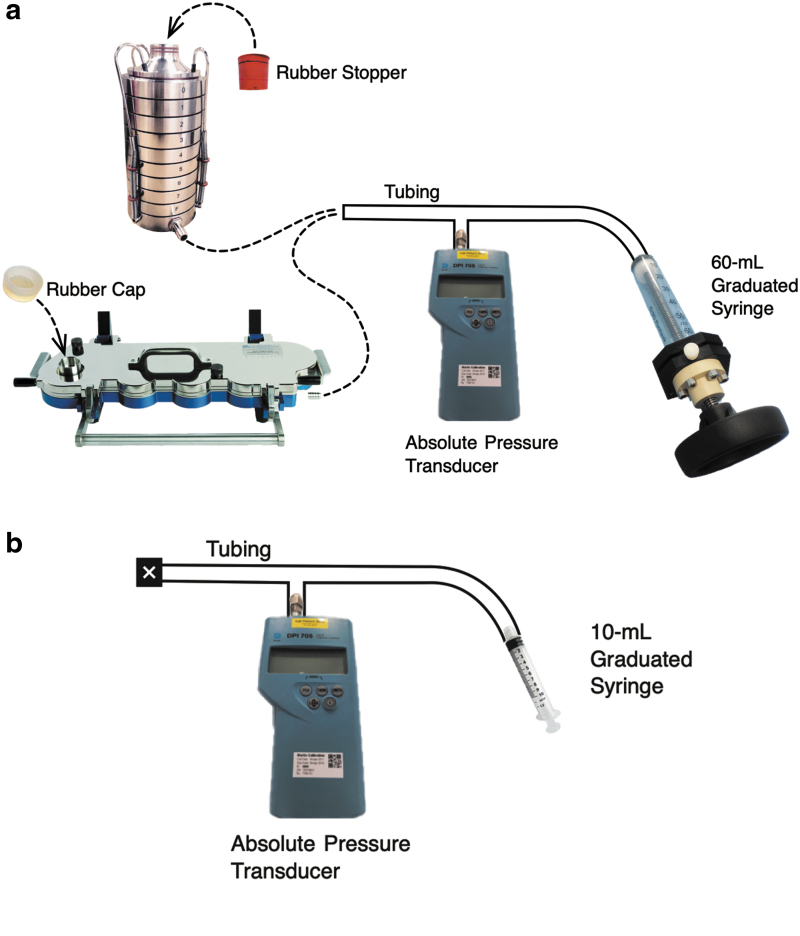
**(a, b)** Arrangement of syringe and pressure transducer for measuring changes in absolute pressure.

The primary method for measuring internal volumes was by following the *P*-*V* behavior in a closed system. In this method, the apparatus-on-test was connected to an ordinary tubing “T-piece” to which necessary measurement equipment could be attached (Fig. 1). This equipment comprised an absolute pressure transducer (Model 705; Druck Ltd., Groby, Leicester, United Kingdom) and an air-tight plastic syringe (Plastipak; Becton-Dickinson and Co., Wokingham, Berkshire, United Kingdom). Repeat measurements were conducted by detaching the syringe from the tubing, pushing the air out of the syringe, and reattaching the tubing. Two syringes were employed with volumes of 60 and 10 mL, graduated in 10- and 1-mL increments, respectively. The 60-mL syringe, with a custom-built threaded plunger shown in Figure 1, was chosen for measuring the volume of the impactors with and without their pre-separators. The 10-mL syringe was chosen for measuring the volume of the measurement apparatus itself with NO impactor components included (i.e., the system “dead” volume).

The method of closing the inlets to each impactor comprised an elastomer cap for the NGI with or without pre-separator and an elastomer stopper for the several ACI configurations. The cap did not occupy any of the NGI internal volume measured. However, the stopper protruded slightly into the inlet of each ACI configuration under evaluation (with either expansion cone or pre-separator as entry to the impactor). The stopper was therefore marked while it was in place, so that the volume of occupied space inside the impactor could be calculated. The stopper occupied ∼3–4 mL for all configurations other than the 90-L/min design with pre-separator. The stopper occupied ∼13 mL for this 90-L/min configuration. These values were added to the measured internal volumes to determine the complete internal volumes.

The routine procedure for measuring the volume of the impactor with or without pre-separator involved recording the pressure with the 60-mL syringe plunger fully inserted, then progressively withdrawing the syringe plunger in increments of 10 mL, and finally recording the stable system pressure at each point. Two sets of tests were performed at different times, a minor factor, but nevertheless for the sake of completeness we have designated the identifiers “set A” and “set B,” when referring to the two different periods. In both sets of data, the system set-ups were identical (i.e., comprising the same connecting tubing with the same pressure transducer). The accuracy of the 60-mL syringe used for data set A was checked by dispensing water into a beaker on a suitably precise gravimetric balance and was found to be within 0.2 mL of the expected value. To determine the apparatus dead volume, the impactor and pre-separator were removed from the test system, and the 10-mL syringe plunger was attached, first fully inserted, and finally withdrawn to volumes of 2, 5, 7, and 10 mL, recording the system pressure at each point.

Three replicate measurements were undertaken at each test condition. Every time the syringe was withdrawn further, thereby changing the internal pressure, the pressure was determined within seconds of executing the withdrawal and then measured again 1 minute later. This procedure allowed real-time quantification of any detectable leak. No leak was detected throughout the test program.

The equation relating *P* and *V* in a closed system, namely P*V = constant, has the form of a rectangular hyperbola, requiring the techniques of nonlinear least-square analysis to deduce the volume when the syringe plunger is set at the reference start condition of zero volume withdrawn (*V*_0_). This volume includes the impactor components and the test tubing and connections (the dead volume). The volume of the impactor components was then determined by subtracting the dead volume (typically 32 mL). The appropriate nonlinear data analysis method for calculating the internal volume of each impactor apparatus is described in detail in Appendix A1. Here, we also describe alternative nonlinear methods, an approximate linearized method, and the error made in the data analysis in the previous study^(10)^; we have compared the results arising from all of these data analysis methods.

The expected internal volume of the compendial induction port by itself was calculated from the nominal dimensions given in the PhEur^(1)^ and also in the USP^(2)^ (details in Appendix A2). The internal volume of one USP/PhEur induction port was also determined experimentally by filling with water and decanting the water into a graduated cylinder for quantification. The goal of the measurement was to determine the volume that is active during an inhaler test (i.e., with the internal surfaces exposed to the passage of aerosol rather than forming part of the coupling to the remainder of the apparatus). Consequently, the design chosen for the testing was the induction port that fits the NGI [MSP Corporation, Shoreview, MN; see fig. 9 of Marple et al.^(13)^]. It is possible with this induction port to determine its internal volume by securing a length of sealing tape tightly to close off one end before slowly filling the other end with water, taking care not to trap air bubbles. The method is referred herein as the “water-fill” technique.

This “water-fill” technique was also used as a check on the *P*-*V* data quality. With the NGI, the “impactor plus pre-separator” volume and the “impactor alone” volume, when measured by *P*-*V* behavior, should differ by the actual volume of the pre-separator. A separate method of measuring the NGI pre-separator volume was devised to test this reasonable expectation. First, the aluminum insert of an NGI pre-separator was removed from the pre-separator, and an elastomer cap was placed on the female-tapered inlet of this “empty” NGI pre-separator. The pre-separator was then placed upside down on a gravimetric balance and filled with water to measure its volume (the upside-down orientation assisted with eliminating air bubbles). The volume of the insert itself was calculated by separately weighing it and dividing by its density (2.70 g/cm^3^). The volume of the insert was then subtracted from the volume of the empty pre-separator to yield the volume of the normal, in-use NGI pre-separator. This test of the data quality could be performed only with an NGI and its pre-separator. An equivalent test with the ACI would have been complicated by the presence of the inlet cone that resides on this impactor design when there is no pre-separator. Also, when this pre-separator was not on the impactor, there was difficulty in either effecting a seal at the connecting end or, if it was inverted, trapping air in the body of the pre-separator.

## Results and Discussion

Tables 1 and 2 list the volumes (mean ± SD, *n* = 3) determined for each impactor apparatus measured, for configurations involving the NGI and the various configurations of the ACI for use at different target flow rates, respectively, and also show the values reported by Copley et al.,^(10)^ where such data are available. The measured volumes were typically 5% smaller than those of the 2005 study; the reason for the divergence is not clear, although the data reduction method employed by Copley et al.^(10)^ can produce this magnitude of discrepancy (Appendix A1). The volumes of several of the ACI configurations were separately assessed by one of the manufacturers (Copley Scientific Ltd., Nottingham, United Kingdom, data not shown), and their computer-aided design (CAD) software values were marginally smaller by no more than 4% compared with the values measured in this study. Given the limitations of the techniques as a whole, these CAD-based values therefore corroborate the current values of internal volume, rather than those reported in 2005. Finally, the uncertainty in the measurements with the closed-system method was in the range from 1% to 2%, a result that conveys confidence in the current data and the importance of using the mathematically correct nonlinear data analysis method that allows for the calculation of the uncertainty in the calculated results (Appendix A1).

**Table 1.** Internal Volumes of Components and Groups of Components Related to Testing with the Next-Generation Impactor for Inhaler Aerosol Aerodynamic Particle Size Distribution

	Component or grouping	Internal volume (mL; current study)	Internal volume (mL; 2005 study)
By individual apparatus	USP/PhEur Induction Port alone	67 ± 1	85
	NGI™ alone	1123 ± 6	1160
	NGI with pre-separator	1855 ± 21	1940^a^
Groups calculated from individual apparatus measurements	NGI with induction port	1172 ± 6	1245
	NGI with induction port and pre-separator	1904 ± 22	2025

^a^ NGI and its pre-separator reported separately by Copley et al.^(10)^

NGI, Next-Generation Impactor; PhEur, European Pharmacopoeia; USP, United States Pharmacopeia.

**Table 2.** Internal Volumes of Components and Groups of Components Related to Testing with the Andersen Cascade Impactor for Inhaler Aerosol Aerodynamic Particle Size Distribution

Measurement approach	Component or grouping	Internal volume (mL; current study)	Internal volume (mL; 2005 study)
By individual apparatus	USP/PhEur induction port alone	67 ± 1	85
	ACI alone; 28.3-L/min configuration (inlet cone, stages 0–7, final filter)	840 ± 8	890
	ACI alone; 60-L/min configuration (inlet cone, stages −1 to 6, final filter)	842 ± 27	Not studied
	ACI alone; 90-L/min configuration (inlet cone, stages −2 to 5, final filter)	845 ± 26	Not studied
	ACI; 28.3-L/min configuration (stages 0–7, final filter) with pre-separator (28.3-L/min version)	1013 ± 7	1070^a^
	ACI; 60-L/min configuration (stages −1 to 6, final filter) with pre-separator (60-L/min version)	1007 ± 5	Not studied
	ACI; 90-L/min configuration (stages −2 to 5, final filter) with pre-separator (90-L/min version)	1053 ± 23	Not studied
Groups calculated from individual apparatus measurements	ACI with induction port	910 ± 8, 27, 26^b^	975
	ACI with induction port and pre-separator	1077 ± 4^c^	1155^d^
		1120 ± 23^e^	

^a^ 28.3-L/min ACI and 28.3-L/min pre-separator reported separately by Copley et al.^(10)^

^b^ Mean value for the three configurations; uncertainties remain for each configuration.

^c^ 28.3- and 60-L/min configurations.

^d^ 28.3-L/min configuration.

^e^ Ninety-liter per minute configuration.

ACI, Andersen cascade impactor.

Other features of the current procedure represent important improvements over the approach taken by Copley et al.^(10)^ In particular, the overall accuracy was improved by making a quantitative measurement of the dead volume of the measurement apparatus itself, a measurement enabled with a syringe with a capacity of just 10 mL. In the previous work, the dead volume had been estimated as consisting only of the sum of the contributions from the tubing making the connections in the system, thereby neglecting the dead volume inside the Druck 705 pressure transducer and the dead space contained in the various connectors themselves. Another refinement was the introduction of a check for leakage of ambient air into the apparatus-on-test at the time each and every data point was acquired. This verification comprised determining the pressure after withdrawing the syringe plunger to a new setting and re-measuring the pressure 1 minute afterward. The pressure reading did not change by more than 0.1% during this 1-minute period, indicating that the requirement of a closed system was maintained throughout the volume determination process. And a final refinement to the method was careful accounting for volume taken up by the rubber stopper used in the testing of the ACI configurations.

The internal volume of the induction port (mean ± SD, *n* = 3) was determined to be 67 ± 1 mL, using the water-filling method. This value agrees with the value of 67.3 mL calculated (Appendix A2) from the nominal dimensions given in the PhEur^(1)^ and the USP.^(2)^ Further investigation revealed that the previously reported internal volume of this component of 85 mL^(10)^ had been based on an early prototype and should therefore no longer be considered representative for this component.

The volume of the NGI pre-separator was determined gravimetrically by filling it with water, and the volume was 804 mL, *absent the insert*. In these water-fill measurements, the NGI pre-separator (PS) was upside down on the gravimetric balance with the female end placed onto an elastomer cap that did not protrude into the pre-separator. In this way, no adjustment had to be made for the volume taken up by a rubber stopper. Water flows easily into the NGI PS male inlet when the PS is upside down, and care was therefore taken not to trap air bubbles near the male taper outlet (which was left open to judge the point when a complete fill was obtained). The insert itself weighed 160.1 g, corresponding to a volume of 59.3 mL (aluminum; density 2.70 g/cm^3^), and yielded an internal volume of 745 mL for the NGI pre-separator as it is normally configured, with the insert located inside. This value agrees within 1% of the volume estimated by subtracting the “NGI alone” result from the “NGI plus pre-separator” result (732 mL). To compare this value of 732 mL with the measured PS volume of 745 mL, the fit of the pre-separator into stage 1 of the NGI has to be considered. The female tapered inlet to stage 1 of the NGI accepts the male tapered outlet of the PS when in use for testing inhaler devices and that is how it was placed for the *P*-*V* testing. The volume of the female-tapered inlet to stage 1 was determined to be ∼18 mL (Appendix A2). Hence, when the NGI PS attaches to the NGI, the expected resulting volume is the PS volume plus the “NGI alone” volume *MINUS* 18 mL. Hence, subtracting the “NGI alone” result from the “NGI with pre-separator” result leads to an estimate of 750 mL for the PS volume. This 750-mL estimate of the internal volume of the PS agrees with the 745 mL measured by the water-fill method, thereby building confidence in the accuracy of the internal volume measurements using the *P*-*V* method.

Table 1 includes a summary of the internal volumes of common arrangements of the components associated with the NGI. The induction port, when added to the NGI added 67 mL, but because the male tapered outlet of the induction port took up the 18-mL volume of the inlet to stage 1, the internal volume of the NGI with its induction port calculates to 1172 mL (67 + 1123 – 18 mL). The corresponding internal volume for the NGI and induction port with its PS is 1904 mL (67 + 1855 – 18 mL). The square root of the sum of the squares of the individual uncertainties was used to estimate the overall uncertainties attributed to these calculated values.

The internal volumes of the three configurations of the ACI (28.3-, 60-, and 90-L/min), when there is a cone on top (no pre-separator), were the same, within the experimental uncertainty (Table 2). At first glance, one might conclude that there is an upward trend (840, 842, and 845 mL). A trend toward greater internal volume might seem logical because of the larger nozzles of the stages in the 60- and 90-L/min configurations [see tables 2-4 and 2-5 of Mitchell and Roberts^(12)^]. However, the stages removed when converting from the 28.3-L/min configuration to the 60-L/min configuration and then to the 90-L/min configuration possess thinner nozzle plates than those added to the top of the Andersen [see table 1 of Vaughan^(14)^], an attribute that decreases the available internal volume. Approximate geometric calculations of these two competing factors support the observed result. Given this measure of agreement, it is recommended that each impactor configuration be regarded as having an internal volume of 840 mL. However, the best practice is to retain the separate uncertainty values reported for each configuration (Table 2), because their magnitudes were found to be different for the three configurations.

When the pre-separator components of the ACI take the place of the inlet cone, the internal volume determined for the 28.3-L/min configuration was the same as that measured for the 60-L/min configuration, within experimental error (Table 2). The listed value for the 28.3-L/min configuration is the average of one point from data set A (1017 ± 11 mL) and one point from data set B (1009 ± 8 mL). The fact that these values for the 28.3-L/min configuration from sets A and B agree within experimental error helps corroborate the method, because the ACI components evaluated for these two data sets were different (same design but different serial numbers), and the individual 60-mL syringes used in the *P*-*V* measurements were different. The pre-separator inlet passageway for the 28.3-L/min configuration is smaller by 8 mL than the inlet passageway for the 60-L/min configuration (Appendix A2). However, the addition of stage −1 and removal of stage 7 (the change from 28.3-L/min configuration to the 60-L/min configuration) decreases the internal volume by less than 10 mL [estimated from Mitchell and Roberts,^(12)^ as noted earlier]. Consequently, geometric considerations support the observation that these two configurations have the same internal volume, within the experimental accuracy.

The 90-L/min configuration with its pre-separator was ∼40 mL larger in internal volume compared with the other two configurations (Table 2). A major portion of this difference can be attributed to the larger internal volume of the pre-separator inlet passageway (∼30 mL larger; Appendix A2). The internal volume of the bottom half of the 90-L/min pre-separator is also slightly larger than that of the 60- or 28.3-L/min version to accommodate the larger dimensions of the 90-L/min inlet component. Finally, the 90-L/min ACI stack was estimated to contain about the same internal volume as the other two configurations. Overall, however, geometric considerations indicate that the 40-mL larger volume of the 90-L/min configuration determined by the *P*-*V* method is reasonably accurate.

Care is needed when interpreting the data contained in Table 2. First, there are three entries where internal volume values are presented for the ACI alone. These impactors, by design, have an entrance cone on top of the stack of size-fractionating stages [fig. 6 and 6b of USP chapter <601>^(2)^]; this cone is removed when the pre-separator is placed on top of the impactor. Therefore, it would be incorrect to subtract the internal volume of the ACI from the internal volume of the ACI with pre-separator and designate the resulting value “the pre-separator volume.” For this reason, no value for “the pre-separator” is given in Table 2. Such a subtraction was the basis of the volume for the pre-separator of the ACI reported by Copley et al.,^(10)^ causing a misleading estimate of the pre-separator volume for this impactor.

**APPENDIX FIG. A2.**
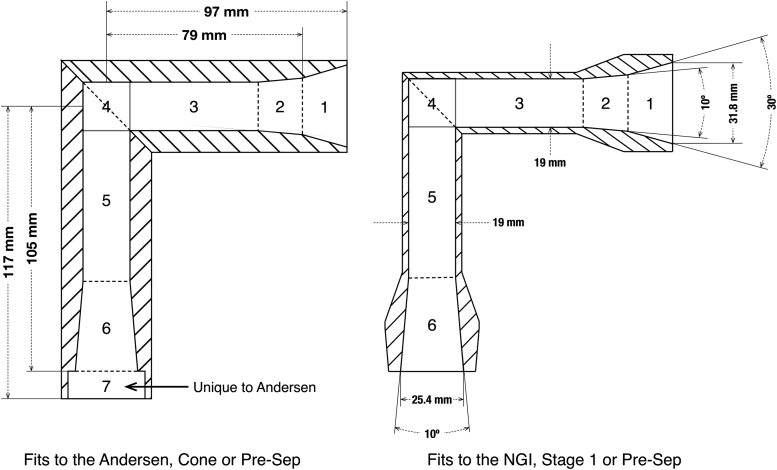
Cross-section of induction port made to fit the ACI (L) and the NGI™ (R)—The internal dimensions of segments 1–6 are identical in both varieties of the induction port; all dimensions shown are given in the USP (independent variables). NGI, Next-Generation Impactor; USP, United States Pharmacopeia.

Second, the USP/PhEur induction port^(2)^ that fits onto the ACI contains ∼9.5 mL of volume in the space that fits onto the ACI [fig. 6a of USP chapter <601>^(2)^; also segment 7 in Appendix Fig. A2]. However, it is important to note that this volume is not available during an inhaler test. Hence, Table 2 reports only 67 mL for the internal volume of the induction port, and only this volume is added to the calculated volumes of the ACI component groups.

Finally, the entire internal volume of a test system, including the inhaler device itself and the tubing and equipment that connects the impactor components to the vacuum source, should be included in any assessment of the entire system internal volume, for example, to evaluate the effect of apparatus internal volume on the rise time to target flow rate in the context of DPI testing. However, since the magnitudes of these additional volumes are user specific, they have not been included in this study.

## Conclusion

The present forensic investigation has provided a definitive set of accurate and precise measures of inhaler aerosol APSD apparatus internal volumes. Water displacement (“water fill method”) was used to determine the internal volume of the USP/PhEur induction port and to test the quality of the *P*-*V* data (NGI with and without pre-separator). The progressive increase in the internal volume of a closed system by syringe plunger movement (*P*-*V* method) was used to measure the more complex corresponding volumes associated with the NGI, both with and without pre-separator, and also for various configurations of the ACI, also with and without pre-separator. A variety of geometric considerations, especially for the induction port, provide reasonable support for the observed experimental results. The data reported herein can be used to define the internal volumes of the various sampling systems described in the pharmacopeial compendia to determine inhaler aerosol APSD that include either cascade impactor design.

## Figures and Tables

**APPENDIX FIG. A1.**
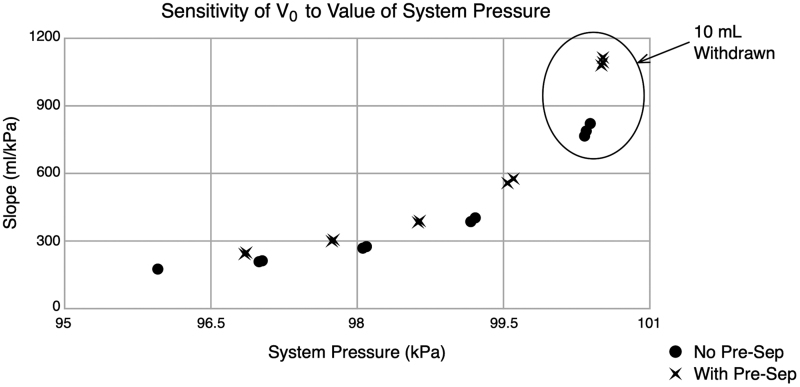
Effect of system pressure on the contribution of individual data points to the uncertainty in *V*_0_ for the 28.3-L/min ACI results.

**APPENDIX FIG. A3.**
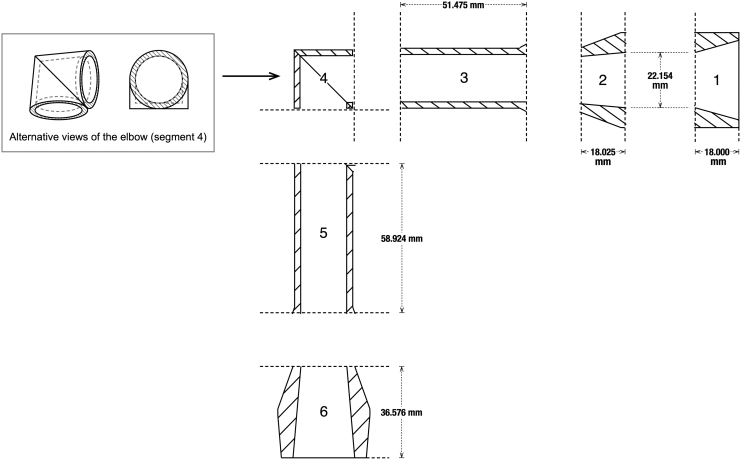
Exploded view of segments of internal volume of the induction port—All dimensions shown are calculated from dimensions given in chapter <601> of the USP (dependent variables).

**APPENDIX FIG. A4.**
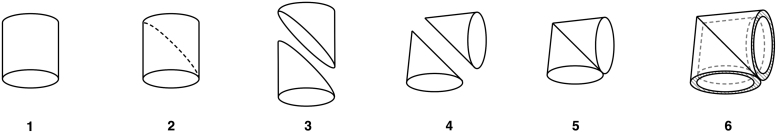
Making an elbow from a cylinder (segment 4)—The internal volume of segment 4; the elbow can be made by taking a cylinder with length equal to its diameter, cutting on a 45° angle through the mid-point, rotating one part by 180°, and joining the two parts at 90°.

**APPENDIX FIG. A5.**
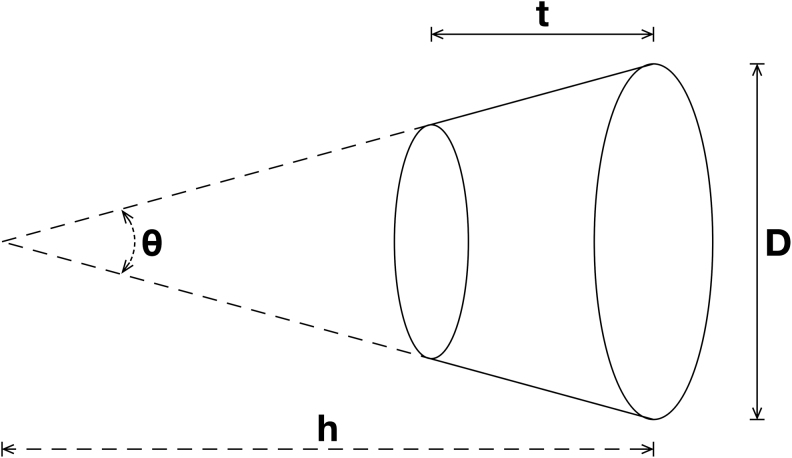
Calculating the volume of a cone segment—The volume of a cone segment is known from the difference in volume of two cones, a larger cone overlaying a smaller cone.

**APPENDIX FIG. A6:**
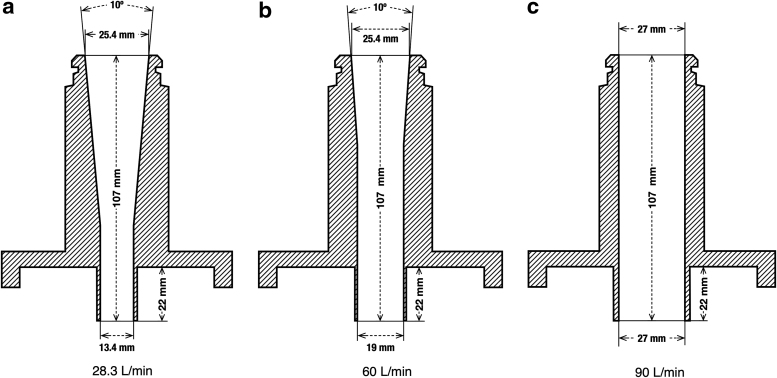
Pre-separator top for three configurations of the ACI.

**APPENDIX FIG. A7.**
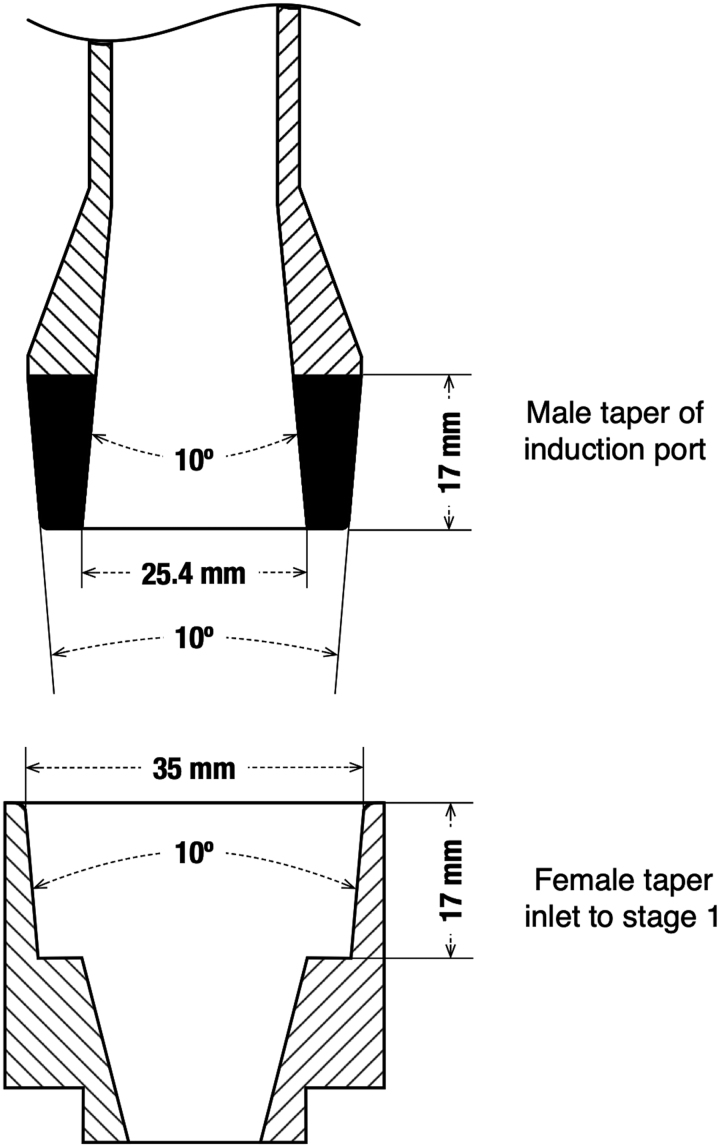
Volume taken up by the male taper of the USP/PhEur induction port when it attaches to the female taper of the entry to the NGI. PhEur, European Pharmacopoeia.

**APPENDIX FIG. A8.**
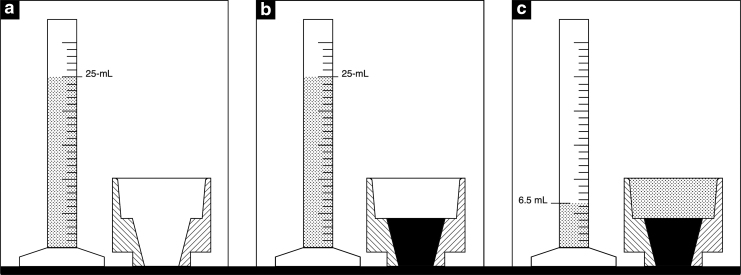
Water-fill determination of volume of female taper of NGI stage 1.**(a)** Measure 25 mL of water into graduated cylinder; **(b)** fill bottom portion of NGI stage 1 with putty; **(c)** add water to fill the top portion of NGI stage 1 and observe remaining volume of water in the graduated cylinder.

## Appendix

### Nomenclature

*P* = absolute pressure inside closed system
*P_i_* = system pressure for data point “i”
*P*_0_ = absolute pressure inside closed system when syringe plunger is at zero
*V* = internal volume of closed system
*V_A_* = additional volume added to the closed system by withdrawal of syringe
*V_A,i_* = additional volume added to the closed system for data point “i”
*V*_0_ = internal volume of closed system when syringe plunger is at zero
*χ* = least-squares minimization parameter

### Approach to the Nonlinear Nature of the Applicable Equations

The ideal gas law applied to a closed system requires that the system pressure times the system volume is a constant. Consequently, if *P*_0_ is the initial pressure in the system and *V*_0_ is the initial volume when the syringe plunger is at zero, then the pressure inside the system, *P*, must decrease as additional volume, *V_A_*, is added by pulling back on the syringe, in accordance with Equation (A-1):

$$P_{0}V_{0}=P*\left(V_{0}+V_{A}\right)$$

Applied to each individual data point, Equation (A-1) becomes:

$$P_{0}V_{0}=P_{i}*\left(V_{0}+V_{A,i}\right)$$

The shape of the curve “P*V = constant” is a rectangular hyperbola and, therefore, linear least-square analyses are inappropriate for the purpose of deducing the best value of *V*_0_ from the experimental data.

Deducing the best-fit parameters from nonlinear relationships between the dependent and independent variables involves consideration of the root philosophy of the mathematical approach. In our analysis, the *P_i_* measurements are the dependent variables, and the values of *V_A,i_* are the independent variables. We assume that the dependent variables exhibit Gaussian statistics, from which least-square theory derives [as explained by Bevington and Robinson^(A1)^]. This approach leads to an equation for the unknown value of *V*_0_, one that must be solved numerically, an outcome that is typical of nonlinear least-square methods. This approach also yields an equation for the uncertainty in the calculated value of *V*_0_.

We will also explain two other nonlinear transformations of the dependent variable that lead to analytical equations for *V*_0_ that are computationally simple. These equations yield values for *V*_0_ that are surprisingly close to those derived from the assumption that the dependent *P_i_* values follow Gaussian statistics, an outcome that must be regarded as “serendipitous,” and that in general depends on the quality of the experimental data.

The following paragraphs explain these three nonlinear methods in detail. We also explain an approximate method that linearizes the relationship between the dependent and independent variables and yields an analytical expression for *V*_0_ by following linear least-square principles. Finally, we explain the method used by Copley et al.^(A2)^ and the error inherent in it. We then show tables with values of *V*_0_ calculated by each of these methods based on the experimental data obtained in this study.

### Method A: Gaussian Statistics and Least-Square Analysis for Nonlinear Equations

Rearranging Equation (A-2) yields a simple relationship between the dependent and independent variables:

$$P_{i}=\frac{P_{0}V_{0}}{V_{0}+V_{A,i}}$$

The dependent variable, *P_i_*, is alone on the left-hand side of Equation (A-3), and the right-hand side is a (nonlinear) function of the independent variable, *V_A,i_*. *P*_0_ is known from the experimental data (atmospheric pressure), and *V*_0_ is the unknown initial system volume, a parameter value that must be estimated from the experimental data. The hypothesis of Gaussian statistics is that if any of the individual tests were to be repeated a large number of times with no change to the independent variable, each measured dependent variable value, *P_i_*, would exhibit a Gaussian distribution whose mean equates to the true value. The assumption of Gaussian statistical probability leads directly to the conclusion that the maximum probable outcome for parameters such as *V*_0_ can be found [chapter 6 of Bevington and Robinson^(A1)^] by minimizing the sum of the square of the difference between the left-hand side and right-hand side of Equation (A-3), evaluated at each data point:

$$\chi_{a}^{2}=\sum {\left(P_{i}-\frac{P_{0}V_{0}}{V_{0}+V_{A,i}}\right)}^{2}$$

(the subscript “a” denotes method A; each method will have its own specific expression for the *χ*^2^ quantity). The value of *V*_0_ that minimizes *χ*^2^ is the best fit value for *V*_0_ and is found by setting the partial derivative of *χ*^2^, with respect to *V*_0_, to zero:

$$\frac{\partial \chi_{a}^{2}}{\partial V_{0}}=0=\sum \left(P_{i}-\frac{P_{0}V_{0}}{V_{0}+V_{A,i}}\right)*\left(\frac{-P_{0}}{V_{0}+V_{A,i}}+\frac{P_{0}V_{0}}{{\left(V_{0}+V_{A,i}\right)}^{2}}\right)$$

After some manipulations, Equation (A-5) can be written as follows:

$$\frac{\sum \frac{P_{i}}{P_{0}}\frac{V_{A,i}}{V_{0}}{\left(1+\frac{V_{A,i}}{V_{0}}\right)}^{-2}}{\sum \frac{V_{A,i}}{V_{0}}{\left(1+\frac{V_{A,i}}{V_{0}}\right)}^{-3}}-1=0$$

Equation (A-6) must be solved numerically for *V*_0_, as is typical for nonlinear least-squares methods. This expression consists of several ratios, all of which are smaller than 1.0, and is purposely written to be dimensionless to facilitate the numerical solution.

The following equation, derived by assuming *V_A,i_*/*V*_0_ is small compared with 1.0, will give a good estimate of *V*_0_, suitable as a first guess in a numerical algorithm, aimed at solving Equation (A-6):

$$V_{0}=\frac{\sum V_{A,i}^{2}}{\sum \left(1-\frac{P_{i}}{P_{0}}\right)*V_{A,i}}$$

In the calculations conducted with the experimental data, the Generalized Reduced Gradient method of the Solver function inside Excel (version 16.29; Microsoft Corporation, Redmond, WA) was used to find a proper value of *V*_0_ that satisfies Equation (A-6), with Equation (A-7) as a starting value. This numerical routine ensured that the calculated value of *V*_0_ caused the absolute value of the left-hand side of Equation (A-6) to be smaller than 10^−7^.

### Method B: A Nonlinear Transformation of the Dependent Variable

As an alternative approach to finding the best value of *V*_0_, we start with Equation (A-2) and re-arrange to find:

$$\frac{P_{0}}{P_{i}}-1=\frac{V_{A,i}}{V_{0}}$$

The left-hand side of Equation (A-8) is a nonlinear function of the dependent variable, *P_i_*, which we call *F*(*P_i_*), for discussion purposes. In method A, we assumed that the dependent variable *P_i_* would exhibit a Gaussian distribution of results, if the experiment were repeated a large number of times. If *P_i_* is assumed to exhibit a Gaussian distribution, *F*(*P_i_*) cannot do so because it is nonlinear in *P_i_*. Therefore, strictly speaking, least-squares analysis will not result in the most probable value of *V*_0_, if we use Equation (A-8) as a starting point. However, if we proceed unabated, the method of least-squares applied to this equation requires finding the value of *V*_0_ that minimizes the following sum:

$$\chi_{b}^{2}=\sum {\left(\frac{P_{0}}{P_{i}}-1-V_{A,i}V_{0}\right)}^{2}$$

The minimum value is found by differentiating with respect to *V*_0_ and setting this derivative equal to zero:

$$\frac{\partial \chi_{b}^{2}}{\partial V_{0}}=0=\sum \left(\frac{P_{0}}{P_{i}}-1-V_{A,i}V_{0}\right)*\left(V_{A,i}V_{0}^{2}\right)$$

Equation (A-10) yields the following expression for *V*_0_:

$$V_{0}=\frac{\sum V_{A,i}^{2}}{\sum \left(\frac{P_{0}}{P_{i}}-1\right)*V_{A,i}}$$

Equation (A-11) is a simple analytical expression for *V*_0_, much easier to calculate than finding the numerical solution to Equation (A-6). It remains to be seen, however, how well it agrees with this expression.

### Method C: Alternative Nonlinear Transformation of the Dependent Variable

Another analytical expression for *V*_0_ can be derived, starting from the following rearrangement of Equation (A-2):

$$\frac{P_{0}V_{0}}{P_{i}}-V_{0}=V_{A,i}$$

As in method B, the left-hand side of Equation (A-12) is a nonlinear function of the dependent variable *P_i_*, and therefore, again, a least-square analysis this time starting with this expression, will not lead to the best value for *V*_0_. Nevertheless, we proceed with this approach, which calls for minimizing the following sum:

$$\chi_{c}^{2}=\sum {\left(\frac{P_{0}V_{0}}{P_{i}}-V_{0}-V_{A,i}\right)}^{2}$$

The value of *V*_0_ that minimizes *χ*^2^ is found by setting the partial derivative of *χ*^2^, with respect to *V*_0_, to zero:

$$\frac{\partial \chi_{c}^{2}}{\partial V_{0}}=0=\sum \left(V_{A,i}-\frac{P_{0}V_{0}}{P_{i}}+V_{0}\right)*\left(1-\frac{P_{0}}{P_{i}}\right)$$

On re-arranging further:

$$0=\sum \left[V_{A,i}*\left(1-\frac{P_{0}}{P_{i}}\right)+V_{0}*{\left(1-\frac{P_{0}}{P_{i}}\right)}^{2}\right]$$

Then, solving for *V*_0_:

$$V_{0}=\frac{\sum V_{A,i}\left(\frac{P_{0}}{P_{i}}-1\right)}{\sum {\left(\frac{P_{0}}{P_{i}}-1\right)}^{2}}$$

### Method D: An Approximate Linearization of the Functional Relationship Between the Dependent and Independent Variables

When *V_A_*/*V*_0_ is much smaller than 1, the right-hand side of Equation (A-2) can be approximated as follows:

$$\frac{P_{i}}{P_{0}}=\frac{1}{1+V_{A,i}V_{0}}\approx 1-V_{A,i}V_{0}$$

In this approximation, a plot of *P_i_* versus *V_A,i_* will have a slope equal to negative *P*_0_/*V*_0_ and an intercept equal to *P*_0_.

It is worthwhile to derive the least-squares fit of this line so that it is possible to see, in quantitative terms, why and to what extent the value of *V*_0_ so calculated differs from the value calculated via Equation (A-6). Ordinary least-squares calculation of *V*_0_ from Equation (A-17) means finding the value of *V*_0_ that minimizes the following sum:

$$\chi_{d}^{2}=\sum {\left(\frac{P_{i}}{P_{0}}-1+V_{A,i}V_{0}\right)}^{2}$$

Minimization occurs when the derivative of this sum with respect to *V*_0_ equals zero:

$$\frac{\partial \chi_{d}^{2}}{\partial V_{0}}=0=\sum \left(\frac{P_{i}}{P_{0}}-1+V_{A,i}V_{0}\right)*V_{A,i}$$

Equation (A-19) can be solved for *V*_0_, and therefore:

$$V_{0}=\frac{\sum V_{A,i}^{2}}{\sum V_{A,i}-\sum \frac{P_{i}V_{A,i}}{P_{0}}}=\frac{\sum V_{A,i}^{2}}{\sum \left(1-\frac{P_{i}}{P_{0}}\right)V_{A,i}}$$

Equations (A-7) and (A-20) are the same, and the former expression is derived from assuming *V_A_*/*V*_0_ is small compared with 1. It is important to appreciate that using Excel to compute the slope and intercept of a plot of *P_i_* versus *V_A,i_* will result in a value for the slope that will give only an approximate value for *V*_0_, no matter the magnitude of the correlation coefficient (*R*^2^) reported. Nevertheless, using the experimental data, it can be shown next that *V*_0_ calculated by Equation (A-20) is larger by about 3%–4% (which equates to between 30 and 40 mL for an apparatus with internal volume close to 1 L, therefore representing a significant over-estimate) from that calculated with nonlinear least-squares with no approximation made [Equation (A-6)].

### Method E: Invalid Approximate Method [as Used by Copley et al.^(A2)^]

In the analysis reported by Copley et al.,^(A2)^ Equation (A-1) was rearranged to yield:

$$V_{A}=\frac{k}{P}-V_{0}$$

Here, *k* is the constant value of *P*_0_ times *V*_0_. On this basis, the quantity “-1*V_0_” is the intercept of the linear plot of *V_A_* versus 1/*P*. From the well-understood equations for a least-squares fit to lines of the form *Y* = *a* + *bX*, Copley et al.^(A2)^ derived the following expression for *V*_0_:

$$\begin{array}{cc} V_{0} & =\frac{\left(\sum \frac{1}{P_{i}}\right)\left(\sum \frac{V_{A,i}}{P_{i}}\right)-\left(\sum {\left(\frac{1}{P_{i}}\right)}^{2}\right)\left(\sum V_{A,i}\right)}{N\sum {\left(\frac{1}{P_{i}}\right)}^{2}-{\left(\sum \frac{1}{P_{i}}\right)}^{2}}\\ & =\frac{\left(\sum \frac{P_{0}}{P_{i}}\right)\left(\sum \frac{P_{0}V_{A,i}}{P_{i}}\right)-\left(\sum {\left(\frac{P_{0}}{P_{i}}\right)}^{2}\right)\left(\sum V_{A,i}\right)}{N\sum {\left(\frac{P_{0}}{P_{i}}\right)}^{2}-{\left(\sum \frac{P_{0}}{P_{i}}\right)}^{2}} \end{array}$$

Here, *N* represents the number of data points in the data set.

The ERROR in this approach is that it ignores the fact that the constant *k* depends on *V*_0_ itself (*k* = *P*_0_*V*_0_). The assumption in the least-square equations [as in Bevington and Robinson^(A1)^], for calculating *a* and *b* in the linear equation *Y* = *a* + *bX* is that *a* does not depend on *b*. And if *a* does depend on *b*, then there is in fact only one unknown constant, not two; and that is the case in the analysis here, because the sole unknown is *V*_0_.

So, Equation (A-22) is incorrect for establishing an accurate value for *V*_0_, and the magnitude of the discrepancy between reported and true values of this metric depends on the experimental data, as shown in what follows.

### Proof That *V*_0_ Calculated by Method B is NOT Identical to That Calculated by Method C

Without careful assessment, it might be plausible to assert that since methods B and C start with essentially the same equation, they should yield the same calculated result for *V*_0_. We can show, however, that Equations (A-11) and (A-16) can never be the same. We start by taking a close look at Equation (A-13) and factoring out the quantity $V_{0}^{2}$:

$$\chi_{c}^{2}=\sum {\left(\frac{P_{0}V_{0}}{P_{i}}-V_{0}-V_{A,i}\right)}^{2}=V_{0}^{2}\sum {\left(\frac{P_{0}}{P_{i}}-1-\frac{V_{A,i}}{V_{0}}\right)}^{2}$$

The summation on the right-hand side of Equation (A-23) is the same as Equation (A-9), therefore

$$\chi_{c}^{2}=V_{0}^{2}*\chi_{b}^{2}$$

Therefore, the following equation relates the derivatives of the *χ*^2^ quantities:

$$\frac{\partial \chi_{c}^{2}}{\partial V_{0}}=2V_{0}*\chi_{b}^{2}+V_{0}^{2}*\frac{\partial \chi_{b}^{2}}{\partial V_{0}}$$

Consequently, when Equation (A-10) is satisfied $\left[\frac{\partial \chi_{b}^{2}}{\partial V_{0}}=0\right]$, Equation (A-14) cannot simultaneously be satisfied $\left[\frac{\partial \chi_{c}^{2}}{\partial V_{0}}=0\right]$, and *vice versa*. Therefore, methods B and C must yield at least slightly different answers for *V*_0_.

### Uncertainty in the Value of *V*_0_

One of the main values of an authentic least-squares fit to the nonlinear Equation (A-1) is the ability to derive an expression for the precision (the uncertainty) of the calculated value of *V*_0_. This undertaking relies on computing the partial derivative of *V*_0_ with respect to *P_i_* so that we know the effect of the uncertainty of each data point on the calculated value for *V*_0_. The following derivation relies on method A because it is the sole proper algebraic solution to the nonlinear least-square equations defining *V*_0_.

Bevington and Robinson^(A1)^ have shown how propagation of error from each data point accumulates to the uncertainty in the calculated parameter(s) in the general case where there are multiple parameters. In the present case, with only one parameter to consider, the following equations define the uncertainty in *V*_0_:

$$\sigma_{V_{0}}^{2}=\sigma_{P}^{2}\sum {\left(\frac{\partial V_{0}}{\partial P_{i}}\right)}^{2}$$

with:

$$\sigma_{P}^{2}=P_{0}^{2}*\frac{1}{N-1}*\sum {\left(\frac{P_{i}}{P_{0}}-\frac{1}{1+V_{A,i}V_{0}}\right)}^{2}$$

Equation (A-27) derives from Equation (A-3); *N* in Equation (A-27) indicates the number of data points (*N* − 1 indicates there is one degree of freedom—namely *V*_0_ itself—in the curve to which the data are being fit).

The partial derivative in Equation (A-26) remains to be calculated. We first re-arrange Equation (A-3) as follows:

$$V_{0}=\frac{V_{A,i}P_{i}}{\left(P_{0}-P_{i}\right)}$$

Differentiating, now, with respect to each measured *P* value, we find:

$$\frac{\partial V_{0}}{\partial P_{j}}=\frac{P_{0}V_{0}}{P_{i}\left(P_{0}-P_{i}\right)}=\frac{P_{0}V_{A,i}}{{\left(P_{0}-P_{i}\right)}^{2}}ifi=j;0ifi\neq j$$

Inserting Equations (A-27) and (A-29) into Equation (A-26), we have:

$$\begin{array}{cc} \sigma_{V_{0}}^{2} & =\left[P_{0}^{2}*\frac{1}{N-1}*\sum {\left(\frac{P_{i}}{P_{0}}-\frac{1}{1+V_{A,i}V_{0}}\right)}^{2}\right]\\ & \sum {\left(\frac{P_{0}V_{0}}{P_{i}\left(P_{0}-P_{i}\right)}\right)}^{2} \end{array}$$

Equation (A-30) can be calculated in a straightforward manner once *V*_0_ itself has been determined from a numerical solution of Equation (A-6). It is helpful to see Equation (A-30) re-arranged in a dimensionless form, as follows:

$$\begin{array}{cc} {\left(\frac{\sigma_{V_{0}}}{V_{0}}\right)}^{2} & =\left[\frac{1}{N-1}*\sum {\left(\frac{P_{i}}{P_{0}}-\frac{1}{1+V_{A,i}V_{0}}\right)}^{2}\right]\\ & \sum {\left(\left(P_{i}P_{0}\right)\left(1-P_{i}P_{0}\right)\right)}^{-2} \end{array}$$

The left-hand side of Equation (A-31) is the relative standard deviation of *V*_0_, and every ratio on the right-hand side is a fraction that is smaller than 1.0.

The impactor internal volume is the system volume measured with the impactor in place minus the system volume measured without the impactor (which we call the “dead” volume), as in Equation (A-32):

$$V_{imp}={\left(V_{0}\right)}_{imp}-{\left(V_{0}\right)}_{dead}$$

Here, *V*_imp_ is the internal volume of the impactor, and the other *V*_0_ terms are the system volume measured with the impactor in place and with the impactor absent. Equation (A-32) is trivial in form; nevertheless, it shows that the resulting uncertainty in *V*_imp_ comes from two sources; hence, Equation (A-33) obtains, yielding the uncertainty in the measured value of the impactor volume:

$$\sigma_{V_{imp}}^{2}=\sigma_{V_{0,imp}}^{2}+\sigma_{V_{0,dead}}^{2}$$

### Method Capability and the Acceptable Range of the *P_i_*/*P*_0_ Ratio

It is important to consider the question of “what values of P_i_” give reasonable values of the uncertainty in *V*_0_. This perhaps unusual question can arise in data analyses when the assumed functional form is nonlinear because each term in the summation in Equation (A-26) depends on the independent variable (in contrast, each term is a constant when the functional form is linear). In the present case, Equation (A-29) is somewhat alarming, because it shows that the sensitivity of the calculated value of *V*_0_ to experimental error is arbitrarily large when the *P_i_* values are nearly equal to the *P*_0_ values. Equation (A-31) also demonstrates that the uncertainty in *V*_0_ grows arbitrarily large when *P_i_* values approach *P*_0_. This result means that if the experimental values of *P_i_* are close to *P*_0_, the uncertainty in the calculated value of *V*_0_ could be so large as to render the calculated value of no value for practical applications.

It is important, then, to quantify an acceptable range of the ratio of *P_i_*/*P*_0_ so that the resulting inherent uncertainty in *V*_0_ is small enough to be of practical use. In fact, finding an acceptable range for the values of *P_i_*/*P*_0_ is part of quantifying whether the method, *P***V* = constant, is capable of producing sufficiently precise values of the impactor internal volumes.

This question of “method capability” must be addressed by quantifying the values of the partial derivatives [left-hand side of Equation (A-29)] at each data point. Appendix Figure A1 depicts representative behavior of these slope values taken from the testing of the 28.3-L/min ACI (Andersen cascade impactor), with and without its pre-separator. Equation (A-26) indicates that the square of the individual slope values is what adds up to the square of the uncertainty in *V*_0_. Therefore, the points on the far right, taken with only 10 mL of volume withdrawn with the syringe, dominate the uncertainty in *V*_0_. This behavior is typical of the entire data set. So, throughout the data analysis, we chose to *NOT USE* the 10-mL data point in the quantification of the mean values and of the uncertainty values for *V*_0_, as reported in the main text in Tables 1 and 2. The mean values, calculated by Equation (A-6) of this Appendix A1, were affected only by fractional milliliters by this neglect of the 10-mL data point, but the uncertainties were reduced by at least 50%.

### Comparing the Several Methods of Calculating *V*_0_

In Appendix Tables A1 and A2, the calculated values of *V*_0_ are listed by using all five data reduction methods (nonlinear methods A, B, C; linearized, approximate method D; invalid method E). Triplicate measurements were taken for each configuration. The three nonlinear, analytical methods agree within 0.01% or better. Of these three methods, only method A properly assigns Gaussian statistical probability to the least-square computations. The fact that all three methods agree rather closely indicates that the experimental data closely follow the functional form of Equation (A-2). A plot of the value of *χ*^2^ versus *V*_0_ as a parameter, for each of the methods A, B, and C, would very likely show that the minima simply converge, even though the functional forms are different.

**Appendix Table A1.** Comparison of *V*_0_ Values Calculated for Next-Generation Impactor Equipment

Impactor configuration	Replicate	Analysis method				
		Nonlinear			Linear, D	Used in 2005, E
		A	B	C		
NGI™ alone	1	1124.000	1123.912	1123.902	1165.361	1113.789
	2	1122.897	1122.886	1122.885	1164.297	1122.333
	3	1122.897	1122.886	1122.885	1164.297	1122.333
NGI with pre-separator	1	1849.583	1849.457	1849.395	1890.986	1816.373
	2	1846.844	1846.776	1846.766	1888.244	1838.061
	3	1867.518	1867.531	1867.517	1909.000	1853.587

NGI, Next-Generation Impactor.

The variability in the data for the 60-L/min ACI with its 60-L/min pre-separator, shown in Appendix Table A2, is greater than in all of the other data sets. Its relative standard deviation, however, is still smaller than 1%, and for that reason, we have not rejected any of these data as “outliers.”

**Appendix Table A2.** Comparison of *V*_0_ Values Calculated for Andersen Cascade Impactor Configurations

Impactor configuration	Replicate	Analysis method				
		Nonlinear			Linear, D	Used in 2005, E
		A	B	C		
28.3-L/min ACI alone	1	838.422	838.369	838.366	879.777	834.517
	2	832.870	833.044	833.027	874.345	844.473
	3	838.741	838.659	838.652	880.081	832.079
28.3-L/min ACI with pre-separator^a^	1	1010.475	1010.494	1010.479	1051.890	1007.886
	2	998.824	998.880	998.874	1040.255	1003.798
	3	1004.006	1003.991	1003.991	1045.395	1002.883
60-L/min ACI alone	1	835.681	835.349	835.145	876.902	817.921
	2	840.689	840.968	840.918	882.212	862.121
	3	837.625	837.947	837.876	879.170	863.688
60-L/min ACI with 60-L/min pre-separator	1	1005.683	1005.732	1005.731	1047.118	1008.143
	2	999.475	999.476	999.475	1040.882	997.310
	3	1003.302	1003.368	1003.362	1044.744	1006.859
90-L/min ACI Alone	1	835.584	835.924	835.850	877.138	862.070
	2	839.283	839.632	839.552	880.843	867.621
	3	838.608	839.066	838.926	880.223	873.045
90-L/min ACI with 90-L/min pre-separator	1	1039.429	1039.677	1039.628	1080.955	1061.956
	2	1038.473	1038.791	1038.707	1080.034	1069.831
	3	1041.254	1041.519	1041.471	1082.800	1064.615

^a^ Data set B, only.

ACI, Andersen cascade impactor.

The linear method (method D) results in values of apparatus internal volume that are typically 3%–4% larger than the true value. The relative simplicity of the linear method is tempting, but it is emphasized that the outcomes are inaccurate.

Finally, method E that was used by Copley et al.,^(A2)^ which erroneously applies a two-parameter method to this one-parameter problem, results in outcomes where the reported apparatus internal volumes are sometimes higher, sometimes lower, and sometimes very close to the true values derived by method A as shown earlier. That behavior is consistent with the incorrect algebra of method E.

As a final comment, the number of significant figures in Appendix Tables A1 and A2 do not reflect experimental precision at all; the three digits to the right of the decimal place exist solely to enable comparison of the outcomes from the data reduction methods. The appropriate precision of each of the measured apparatus volumes is given in the main text.

### Nomenclature

*D* = diameter of larger end of cone segment
*H* = height of a cone
*R* = radius of base of a cone
*V* = volume of a cone
*V_L_* = volume of larger of two cones created by extending sides of cone segment
*V_s_* = volume of smaller of two cones created by extending sides of cone segment
*V*_segment_ = volume of cone segment
*h* = height of cone derived by extending sides of cone segment
*t* = thickness of cone segment
θ = total included angle between sides of cone segment

### The United States Pharmacopeia/European Pharmacopoeia Induction Port

Appendix Figure A2 shows the nominal dimensions of the United States Pharmacopeia (USP)/European Pharmacopoeia (PhEur) Induction Port that are important for calculating its internal volume (Appendix Table A3). There are several logical segments to the induction port, and these are numbered 1–7. When in use, the internal volume of the induction port consists of segments 1–6. So, to calculate the internal volume of the induction port, we ignore segment 7 (volume of 9.5 mL), because that segment has to do solely with attaching to the ACI.

**Appendix Table A3.** Dimensions and Volumes of Segments of United States Pharmacopeia/European Pharmacopoeia Induction Port

Segment	Shape type	Larger base (mm)	Smaller base (mm)	Total included angle (degrees)	Segment thickness (mm)	Volume (mL)
1	Cone segment	*31.800*	22.154	*30*	18.000	10.398
2-	Cone segment	22.154	*19.000*	*10*	18.025	6.005
*Segment*	*Shape type*	*Length (mm)*	*Diameter (mm)*		*Volume (mL)*	
3	Cylinder	51.475	*19.000*		14.595	
4	Cylinder	*19.000*	*19.000*		5.387	
5	Cylinder	58.924	*19.000*		16.707	
*Segment*	*Shape type*	*Larger base (mm)*	*Smaller base (mm)*	*Total included angle (degrees)*	*Segment thickness (mm)*	*Volume (mL)*
6	Cone segment	*25.400*	*19.000*	*10*	36.576	14.256

Entries in italics are values given in the USP,^(A3)^ the independent variables; values in normal font are dependent variables, calculated from the independent variables and simple geometric principles, some of which are explained in the text.

USP, United States Pharmacopeia.

Appendix Figure A3 is an exploded view of the six segments of the induction port that are necessary for calculating the internal volume along with some helpful dimensions that can be calculated from the dimensions given in the USP. Segments 1, 2, and 6 are portions of cones. Segments 3 and 5 are cylinders. The internal volume of segment 4, the elbow, might seem elusive at first glance. However, this elbow can be made by taking a cylinder with length equal to its diameter, cutting on a 45° angle through the mid-point, rotating one part by 180°, and then joining the two parts at 90° (Appendix Fig. A4). Therefore, the volume of the elbow is that of a cylinder with its length equal to its diameter.

Appendix Table A3 gives the volumes of each segment along with the key dimensions, both the independent dimensions given in chapter <601>of the USP^(A3)^ and the dependent dimensions calculated from those given in this source. The total internal volume of the induction port is 67.348 mL. Assuming one-half of the elbow can be associated with the horizontal portion of the induction port, the horizontal section has an internal volume of 33.692 mL, and the vertical section has an internal volume of 33.656 mL. It should be recognized that manufactured induction ports are not going to have these exact dimensions.

It is a straightforward exercise to calculate the volume of each cone segment, but it is helpful to note that the volume of a cone segment is known from the difference in volume of two cones, a larger cone overlaying a smaller cone, as in Appendix Figure A5. Any cone of height *H* and a base with radius *R* has a volume given by the following expression:

$$V=\frac{\pi }{3}*R^{2}*H$$

Consequently, from Equation (A-34), the volume of the cone in Appendix Figure A5 that is formed by the larger base, expressed in terms of base diameter, *D*, is given by:

$$V_{L}=\frac{\pi }{3}{\left(\frac{D}{2}\right)}^{2}*h=\frac{\pi }{3}{\left(\frac{D}{2}\right)}^{2}\frac{\left(D2\right)}{tan\left(\frac{\theta }{2}\right)}=\frac{\pi }{3}{\left(\frac{D}{2}\right)}^{3}cot\left(\frac{\theta }{2}\right)$$

The volume of the cone formed by the smaller base is given by:

$$\begin{array}{cc} V_{s} & =\frac{\pi }{3}{\left(\frac{b_{2}}{2}\right)}^{2}*\left(h-t\right)=\frac{\pi }{3}{\left(\left(h-t\right)tan\left(\frac{\theta }{2}\right)\right)}^{2}\left(h-t\right)\\ & =\frac{\pi }{3}{\left(\frac{D}{2}\right)}^{3}cot\left(\frac{\theta }{2}\right){\left(1-\frac{t}{h}\right)}^{3} \end{array}$$

with:

$$h=\frac{D}{2}cot\left(\frac{\theta }{2}\right)$$

In Equation (A-36), we have denoted the thickness of the cone segment by *t* and the diameter of the smaller cone base by *b*_2_.

Therefore, the cone segment volume is given by:

$$V_{segment}=V_{L}-V_{s}=\frac{\pi }{3}{\left(\frac{D}{2}\right)}^{3}cot\left(\frac{\theta }{2}\right)\left[1-{\left(1-\frac{t}{h}\right)}^{3}\right]$$

In deriving Equation (A-38), it is not necessary to calculate the diameter of the smaller end of the cone segment, denoted as *b*_2_ in Equation (A-36). However, because in some of the cone segments of the USP induction port, the independent variables include the diameter of the smaller end of the cone segment, it is worthwhile to note that the diameter of the smaller end is related to the diameter of the larger end as follows:

$$b_{2}=D-2t*tan\left(\frac{\theta }{2}\right)$$

### Inlet Passageway of the Andersen Pre-separator

The pre-separator that fits onto the ACI has a top piece and a base. The top piece has a passageway through which air enters the pre-separator, and we call this passageway the “inlet passageway.” Appendix Figure A6 depicts the key dimensions of these top pieces, as designed for the flow rates of 28.3, 60, and 90 L/min. The internal volume of these inlet passageways varies from 26.3, 34.2, and 61.3 mL, simply based on the geometry. The ∼30-mL difference between the 90-L/min version and the others is largely responsible for the larger observed internal volume of the 90-L/min ACI when tested with its pre-separator, as mentioned in the main text.

### The Internal Volume of the Female Taper of the Next-Generation Impactor Stage 1 and of the Inlet to Its Pre-separator

When the male taper on the induction port slips into the female taper of the Next-Generation Impactor (NGI™) stage 1 or into the female-tapered inlet to the pre-separator, it occupies a volume that otherwise is empty. In this section, we estimate this volume. This same volume is occupied by the male taper of the pre-separator exit when it attaches to stage 1.

Stage 1 of the NGI and of the female inlet of the NGI pre-separator have a taper consisting of a 10° total included angle.^(A4)^ Exact dimensions of the female inlet to stage 1 or to the pre-separator are not published, but simple measurements showed a height of 17 mm and a diameter of 35 mm (Appendix Fig. A7). The blackened end of the induction port is the portion of the induction port that drops into the female taper when the induction port attaches to stage 1 or to the female taper of the NGI pre-separator inlet. The internal shape of the female taper of stage 1 is a cone segment, and the volume of it can be calculated with the equations given earlier. With the 35-mm wide diameter at the top, the 17-mm height, and the 10° angle, the volume is calculated to be 18.1 mL. We also measured this volume by filling the base of stage 1 with putty up to the point of the 10° taper (solid black; Appendix Fig. A8). Then, we decanted water from a graduated cylinder until the female taper was full (dotted area; Appendix Fig. A8). The volume of water delivered was 18.5 mL. So, for purposes of calculating or comparing the volume of arrangements that include placing the male taper of the induction port (same male taper at the outlet of the NGI pre-separator) into the female inlet of the pre-separator or stage 1 of the NGI, we accounted for this volume as 18 mL, as explained in the main text.

## Notes

Reviewed by:

Keith Ung

Anthony Hickey
